# Assembly and comparative analysis of the complete mitochondrial genome of *Bupleurum chinense* DC

**DOI:** 10.1186/s12864-022-08892-z

**Published:** 2022-09-21

**Authors:** Yonggang Qiao, Xinrui Zhang, Zheng Li, Yun Song, Zhe Sun

**Affiliations:** College of Life Sciences, Shanxi Agricultural University, Taigu, 030801 Shanxi China

**Keywords:** *Bupleurum chinense*, Mitochondrial genome, Repeat sequence, Phylogenetic analysis

## Abstract

**Background:**

*Bupleurum chinense*(*B. chinense)* is a plant that is widely distributed globally and has strong pharmacological effects. Though the chloroplast(cp) genome of *B. chinense* has been studied, no reports regarding the mitochondrial(mt) genome of *B. chinense* have been published yet.

**Results:**

The mt genome of *B.chinense* was assembled and functionally annotated. The circular mt genome of *B. chinense* was 435,023 bp in length, and 78 genes, including 39 protein-coding genes, 35 tRNA genes, and 4 rRNA genes, were annotated. Repeat sequences were analyzed and sites at which RNA editing would occur were predicted. Gene migration was observed to occur between the mt and cp genomes of *B. chinense* via the detection of homologous gene fragments. In addition, the sizes of plant mt genomes and their GC content were analyzed and compared. The sizes of mt genomes of plants varied greatly, but their GC content was conserved to a greater extent during evolution. Ka/Ks analysis was based on code substitutions, and the results showed that most of the coding genes were negatively selected. This indicates that mt genes were conserved during evolution.

**Conclusion:**

In this study, we assembled and annotated the mt genome of the medicinal plant *B. chinense*. Our findings provide extensive information regarding the mt genome of *B. chinense*, and help lay the foundation for future studies on the genetic variations, phylogeny, and breeding of *B. chinense* via an analysis of the mt genome.

## Background

*Bupleurum chinense* DC. is a perennial herb belonging to the Umbelliferae family [1]. Approximately 200 species of Bupleurum are distributed worldwide. *Bupleurum* L. has been used as a medicinal material in China for many years. The Chinese Pharmacopoeia states that the *B. chinense* and *B. scorzonerifolium* species are mainly used as drugs [2]. *B. chinense* is mainly grown in North and Northwest China. It is also distributed in relatively smaller amounts in other regions. The main components of *B. chinense* are saikosaponins, sterols, volatile oils, fatty acids, and polysaccharides [3]. These components have anti-pyretic, anti-inflammatory, and immune functions, and pharmacological effects that prevent liver injury [4–6]. In recent years, the pharmacological functions of *B. chinense* have been explored continuously, because of which it is currently considered as a natural resource with important economic and medicinal value [7, 8].

Mitochondria are important organelles in plant cells that participate in many metabolic processes related to the synthesis and degradation of intracellular compounds and energy production [9, 10]. Endosymbiosis origin theory states that mitochondria originate from endosymbiotic bacteria that can carry out aerobic respiration and are phagocytosed by primitive eukaryotic cells. After the bacteria are swallowed by primitive eukaryotic cells, they gradually evolve into organelles with specific functions in the long-term mutually beneficial process of symbiosis [11–14]. Mitochondria are semi-autonomous, possess relatively independent genetic material, and have an independent and self-sufficient system for protein synthesis [15, 16]. As an energy factory in the cell, mitochondria provide energy for intracellular biosynthesis and degradation. They represent the main site at which cells carry out aerobic respiration and participate in various life activities, such as intracellular differentiation, apoptosis, growth, and division [17–19]. Therefore, the mt genome is a valuable source of genetic information for the study of plant phylogeny and necessary cellular processes, and is of great significance in the study of species evolution, species identification, and genetic transformation [20, 21].

There are significant differences in the length, gene sequence, and gene content of plant mt genomes. This phenomenon is observed not only in different species, but also in the same species [22–24]. Some researchers found Double-strand break repair processes drive evolution of the mt genome in *Arabidopsis*, gene conversion and mismatch repair activity observed in the mt genome of the *Arabidopsis* mutants [22]. With the continuous development of sequencing technology, the mt genomes of a variety of plants have been published [25–27]; however, no report on the *B. chinense* mt genome has been published yet. In this study, we sequenced and annotated the *B. chinense* mt genome and conducted a thorough analysis with regard to genomic characteristics, repetitive sequences, RNA editing, codon preference, and comparative genomics. We performed system evolution analysis to understand the genetic variations in *B. chinense* more effectively, along with reports regarding breeding and plant research on *B. chinense*, as this would provide a theoretical foundation for conducting further research.

## Results

### Genomic features of the *B. chinense* mt genome

**Fig. 1:**
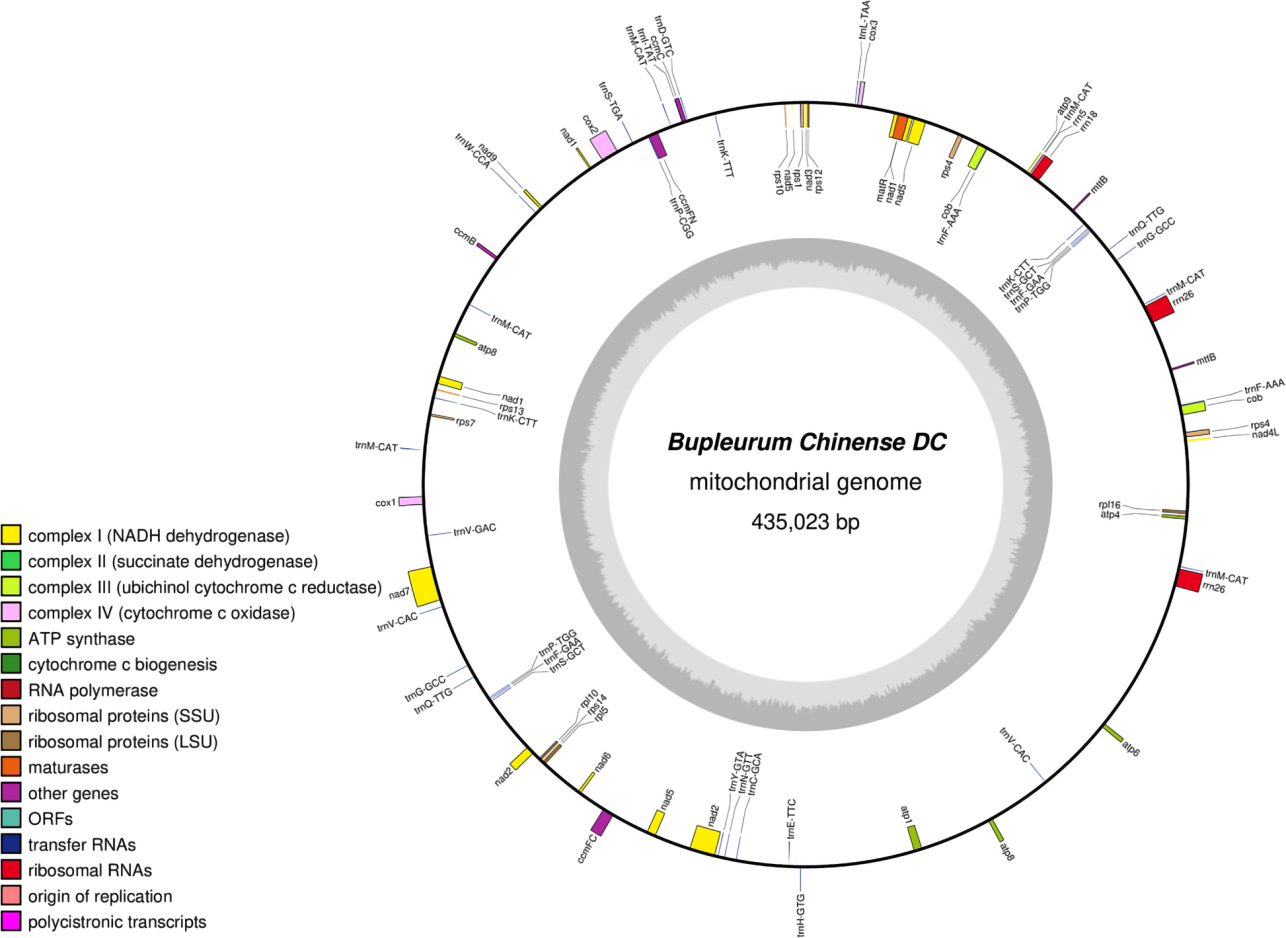
The circular map of the *B. chinense* mt genome

The *B. chinense* mt genome is a circular sequence with a length of 435,023 bp. The genome is composed of the A (27.19%), G (22.49%), T (27.77%), and C (22.55%) bases. The GC content is 45.04%. The functional classification and physical location of the annotated genes are shown in Fig. 1. 78 genes, including 39 protein-coding genes, 35 tRNA genes, and 4 rRNA genes, were annotated in the mt genome. We identified 2283 open reading frames (ORFs).

The results of this process are shown in Table 1. The *B. chinense* mt genome encodes 35 different proteins, which can be divided into 9 categories, and it contains two copies of *nad7, cob*, *rps4*, and *mttB*. The encoded proteins can be classified as NADH dehydrogenases (9 genes), ATP synthases (6 genes), cytochrome C biogenesis accessory proteins (4 genes), cytochrome C oxidases (3 genes), maturases (1 gene), ubiquinol cytochrome c reductases (1 gene), ribosomal proteins (SSU) (7 genes), ribosomal proteins (LSU) (3 genes), and transport membrane proteins (1 gene). The start codon of all protein-coding genes was ATG, and the use rates of the TAA, TGA, and TAG stop codons were different. The use rates of TAA, TGA, and TAG were 51.3, 25.6, and 23.1%, respectively. The use rate of the TAA stop codon was the highest.

**Table 1:** Functional classification of genes and physical location of the *B. chinense* mt genome

Group of genes	Gene name	Length(bp)	Start codon	Stop codon	Amino acid
NADH dehydrogenase	*nad1*	978	ATG	TAA	326
	*nad2*	1467	ATG	TAA	489
	*nad3*	357	ATG	TAA	119
	*nad4*	1488	ATG	TGA	496
	*nad4L*	303	ATG	TAA	101
	*nad5*	2013	ATG	TAA	671
	*nad6*	618	ATG	TAA	206
	*nad7(2)*	1185	ATG	TAG	395
	*nad9*	573	ATG	TAA	191
ATP synthase	*atp1*	1527	ATG	TGA	509
	*atp4*	579	ATG	TAA	193
	*atp6*	795	ATG	TGA	265
	*atp8*	690	ATG	TAG	230
	*atp8*	822	ATG	TAG	274
	*atp9*	225	ATG	CAA(TAA)	75
Cytochrome c biogenesis	*ccmB*	621	ATG	TGA	207
	*ccmC*	753	ATG	TGA	251
	*ccmFc*	1317	ATG	CGA(TGA)	439
	*ccmFn*	1740	ATG	TGA	580
Cytochrome c oxidase	*cox1*	1497	ATG	TAA	499
	*cox2*	795	ATG	TAA	265
	*cox3*	798	ATG	TGA	266
Maturases	*matR*	1968	ATG	TAG	656
Ubiquinol cytochrome c reductase	*cob(2)*	1695	ATG	TAG	565
Ribosomal proteins (LSU)	*rpl10*	462	ATG	TGA	154
	*rpl16*	516	ATG	TAA	172
	*rpl5*	555	ATG	TAA	185
Ribosomal proteins (SSU)	*rps1*	606	ATG	TAA	202
	*rps10*	330	ATG	TAA	110
	*rps12*	378	ATG	TGA	126
	*rps13*	351	ATG	TAA	117
	*rps14*	303	ATG	TAA	101
	*rps4(2)*	918	ATG	TAA	306
	*rps7*	447	ATG	TAA	149
Transport membrane protein	*mttB(2)*	375	ATG	TAG	125
Ribosomal RNAs	*rrn18*	1764			
	*rrn26(2)*	3252			
	*rrn5*	117			
Transfer RNAs	*trnC-GCA*	71			
	*trnD-GUC*	74			
	*trnE-UUC*	72			
	*trnF-AAA(2)*	(85,85)			
	*trnF-GAA(2)*	(74,74)			
	*trnG-GCC(2)*	(72,72)			
	*trnH-GUG*	74			
	*trnI-UAU*	76			
	*trnK-CUU(2)*	(73,73)			
	*trnK-UUU*	73			
	*trnL-UAA*	70			
	*trnM-CAU(6)*	(73,73,77,73,73,73)			
	*trnN-GUU*	72			
	*trnP-CGG*	88			
	*trnP-UGG(2)*	(75,75)			
	*trnQ-UUG(2)*	(72,72)			
	*trnS-GCU(2)*	(88,88)			
	*trnS-UGA*	87			
	*trnV-CAC(3)*	(72,72,72)			
	*trnW-CCA*	74			
	*trnY-GUA*	83			

Numbers after gene names are the number of copies

Studies have shown that the mt genome of most terrestrial plants contains 3 rRNA genes [11, 13]. Here, 3 rRNA genes from the *B. chinense* mt genome, namely *rrn18* (1764 bp), *rrn26* (3252 bp), and *rrn5* (117 bp), were annotated. In addition, 21 different tRNAs, which were involved in the transportation of a total of 17 amino acids, were identified in the *B. chinense* mt genome. This could be explained by the fact that two or more tRNAs might transport the same amino acid to different codons. For example, *trnF-AAA* and *trnF-GAA* are associated with the synonymous codons UUU and UUC, which are involved in the transportation of phenylalanine.

### Repeat sequence analysis

**Fig. 2:**
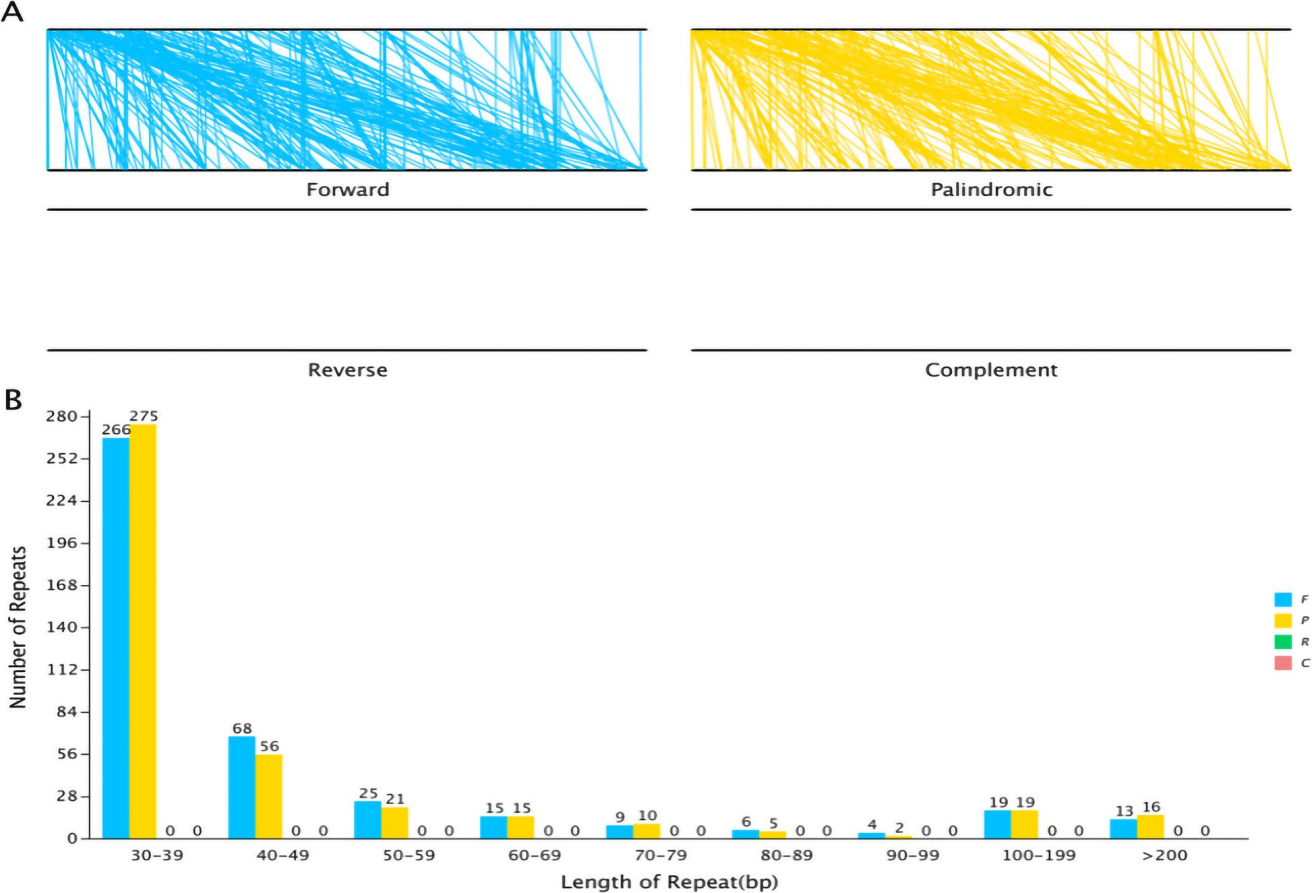
The interspersed repeat sequences in the *B. chinense* mt genome. **A** The four interspersed repeat types are distributed throughout the genome; the two black lines represent the mt genome, and the same repeats are associated with the line segments. **B** Distribution of lengths of interspersed repeats in the mt genome. The abscissa indicates the type of interspersed repeats, and the ordinate indicates the number of scattered repeats

Interspersed repeat sequences are repetitive sequences that are scattered in the genome. In the *B. chinense* mt genome, we identified a total of 844 interspersed repeats with a length greater than or equal to 30 bp; of these, 425 were forward repeats and 419 were palindrome repeats. The length of the longest forward repeat sequence was 12,012 bp and that of the longest palindrome repeat sequence was 16,761 bp. The distribution of the lengths of forward repeats and palindrome repeats is shown in the Fig. 2; the abundance of both types of repeats was the highest when repeats were in the range of 30-39 bp.

Microsatellites, also known as simple sequence repeats (SSRs), are DNA fragments with a length of 1-6 bp [28]. Microsatellites are widely used in species research due to their advantages, which include their polymorphism, codominant inheritance, relative abundance, and wide genome coverage [29, 30]. As shown in Table 2, in the *B. chinense* mt genome, the detected SSR sites included monomer, dimer, trimer, tetramer, pentamer, and hexamer repeats. Among these, the number of monomer repeats was the largest, and accounted for 47.75% of the total SSRs, followed by dimer repeats, which accounted for 36.75% of the total SSRs; the number of pentamer and hexamer repeats was the least. Monomer repeats composed of A/T bases accounted for 91.6% of monomer SSRs, and dimer repeats composed of AG/CT bases accounted for 59.2% of dimer SSRs.

**Table 2:** Distribution of SSRs in the *B. chinense* mt genome

SSR type	Repeats	Number of SSRs	Total
monomer	A/T	175	191
	C/G	16	
dimer	AC/GT	11	147
	AG/CT	87	
	AT/AT	42	
	CG/CG	7	
trimer	AAC/GTT	3	16
	AAG/CTT	7	
	AAT/ATT	3	
	ACT/AGT	2	
	AGG/CCT	1	
tetramer	AAAC/GTTT	1	35
	AAAG/CTTT	16	
	AAAT/ATTT	1	
	AACC/GGTT	2	
	AAGC/CTTG	4	
	AAGT/ACTT	1	
	AATC/ATTG	1	
	AATG/ATTC	3	
	ACTC/AGTG	1	
	AGCC/CTGG	1	
	AGCT/AGCT	2	
	ATCC/ATGG	1	
	CCCG/CGGG	1	
pentamer	AAAAG/CTTTT	1	7
	AAAAT/ATTTT	1	
	AAAGT/ACTTT	1	
	AAGCC/CTTGG	1	
	ACCCG/CGGGT	1	
	ACTAG/AGTCT	1	
	CCCGG/CCGGG	1	
hexamer	AAAGAT/ATCTTT	2	4
	AAATAG/ATTTCT	1	
	AGCCCT/AGGGCT	1	

Tandem repeat sequences, also known as satellite DNAs, refer to repetitive sequences formed by the association between short sequences with 1 to 200 bases as repeating units in tandem, and are widely present in eukaryotic genomes and some prokaryotes [31, 32]. Tandem Repeats Finder v4.09 [33] was used to identify tandem repeats in the *B. chinense* mt genome. As shown in Table 3, a total of 10 tandem repeats ranging in length from 9 to 71 bp that had a match degree greater than 95% were found in the genome.

**Table 3:** Distribution of tandem repeats in the *B. chinense* mt genome

No.	Size	Repeat Sequence	Copy	Percent Matches	Start	End
1	12	GCGCCTGAGCCA	5.3	100	14,015	14,078
2	12	GCGCTGGCTCAG	5.3	100	74,820	74,883
3	12	AATAATAGATAT	2.1	100	133,437	133,461
4	30	AGTAAGTAAACTACCTCTCCTACCTAGTCG	5.5	100	186,768	186,933
5	30	GTAAACTACCTCTCCTACCTAGTCGAGTAA	3.4	98	241,705	241,805
6	34	CACTTAGCTCTTATTGCGACATTCCTTGATATGG	2.1	100	292,143	292,214
7	29	TTGAAGCTTTGCCAGGAAGCTTCTACTTG	2.4	100	325,136	325,204
8	9	TCATAAATC	3.0	100	337,032	337,058
9	71	TATATGAAACGAAAACATTCGTTCAAGTTATAG CTTTCCGGTAAGGAAATGGCATTGCTAGCTTTT CCATA	2.3	96	347,890	348,051
10	30	AGGTAGTTTACTTACTCGACTAGGTAGGAG	3.4	98	371,343	371,443

### Prediction of RNA editing sites

**Fig. 3:**
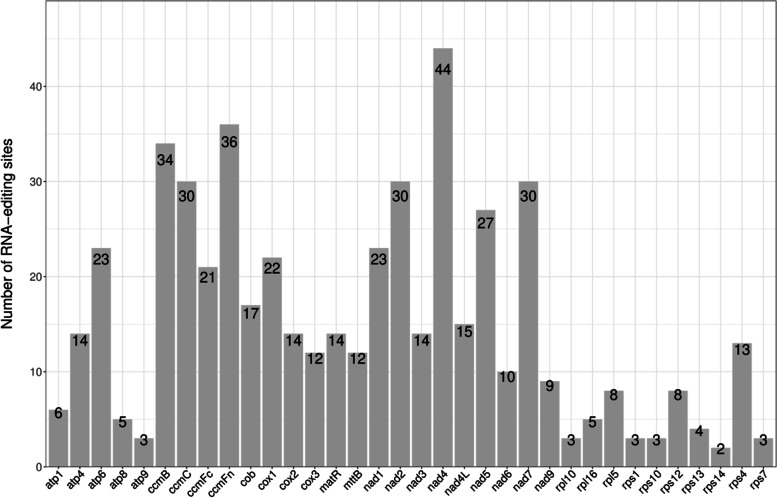
Distribution of RNA editing sites among *B. chinense* mt protein-coding genes

In all eukaryotes, the addition, loss, or substitution of bases in the coding region of the transcribed RNA is called RNA editing [34, 35]. In the mt and chloroplast genomes of plants, RNA editing is manifested as the conversion of specific cytosines to uracils, and it changes the genetic information in the genome [36]. In this study, RNA editor (PREP) [37] (http://prep.unl.edu/) was used to predict sites at which RNA editing would occur. A total of 517 RNAs were predicted in 34 protein-coding genes (Table 4) of the *B. chinense* mt genome (Fig. 3). Among the editing sites, the ribosomal protein (SSU) encoding gene *rps14* contained the least predicted RNA editing sites, i.e., 2 sites, and the NADH dehydrogenase encoding gene *nad4* contained the most predicted editing sites, i.e., 44 sites. After RNA editing, the hydrophobicity of 42.75% of amino acids remained unchanged, while 8.12% of hydrophobic amino acids became hydrophilic, and 48.16% of hydrophilic amino acids became hydrophobic. All RNA-editing sites in the *B. chinense* mt genome are the C-T editing type, among these, 30.95% (160) of the editing sites were located on the first base of the triplet codon, and 65.18% of the editing sites were located on the second base of the triplet codon (337). At certain instances, both the first and second bases of the triplet codon were edited. This caused the conversion of proline (CCC) to phenylalanine (TTC, TTT). RNA editing not only leads to changes in the encoded amino acids, but may also lead to the premature termination of the coding process [38]. In the *B. chinense* mt genome, this phenomenon could be observed in the coding genes *atp6*, *atp9*, *ccmFc*, *cob*, and *rpl16*. The predicted results also show that the amino acids generated after codon editing had the highest tendency to convert to leucine after RNA editing; 43.91% (227 positions) of amino acids were converted to leucine, and the second-highest number of amino acids were converted to phenylalanine, and this accounted for 23.40% of all conversions (121 sites).

**Table 4:** Prediction of RNA editing sites

Type	RNA-editing	Number	Percentage
hydrophobic	CCA (P) = > CTA (L)	43	30.56%
	CCC (P) = > CTC (L)	7	
	CCC (P) = > TTC (F)	6	
	CCG (P) = > CTG (L)	39	
	CCT (P) = > CTT (L)	22	
	CTC (L) = > TTC (F)	6	
	CTT (L) = > TTT (F)	12	
	GCC (A) = > GTC (V)	1	
	GCG (A) = > GTG (V)	6	
	GCT (A) = > GTT (V)	2	
	CCT (P) = > TTT (F)	14	
hydrophilic	CAC (H) = > TAC (Y)	6	12.19%
	CAT (H) = > TAT (Y)	13	
	CGC (R) = > TGC (C)	13	
	CGT (R) = > TGT (C)	31	
hydrophobic-hydrophilic	CCA (P) = > TCA (S)	10	8.12%
	CCC (P) = > TCC (S)	13	
	CCT (P) = > TCT (S)	16	
	CCG (P) = > TCG (S)	3	
hydrophilic-hydrophobic	ACA (T) = > ATA (I)	3	48.16%
	ACC (T) = > ATC (I)	2	
	ACG (T) = > ATG (M)	9	
	ACT (T) = > ATT (I)	4	
	CGG (R) = > TGG (W)	32	
	TCA (S) = > TTA (L)	77	
	TCC (S) = > TTC (F)	32	
	TCG (S) = > TTG (L)	39	
	TCT (S) = > TTT (F)	51	
hydrophilic-stop	CAA (Q) = > TAA (X)	3	0.97%
	CAG (Q) = > TAG (X)	1	
	CGA (R) = > TGA (X)	1

### Analysis of codon composition

**Fig. 4:**
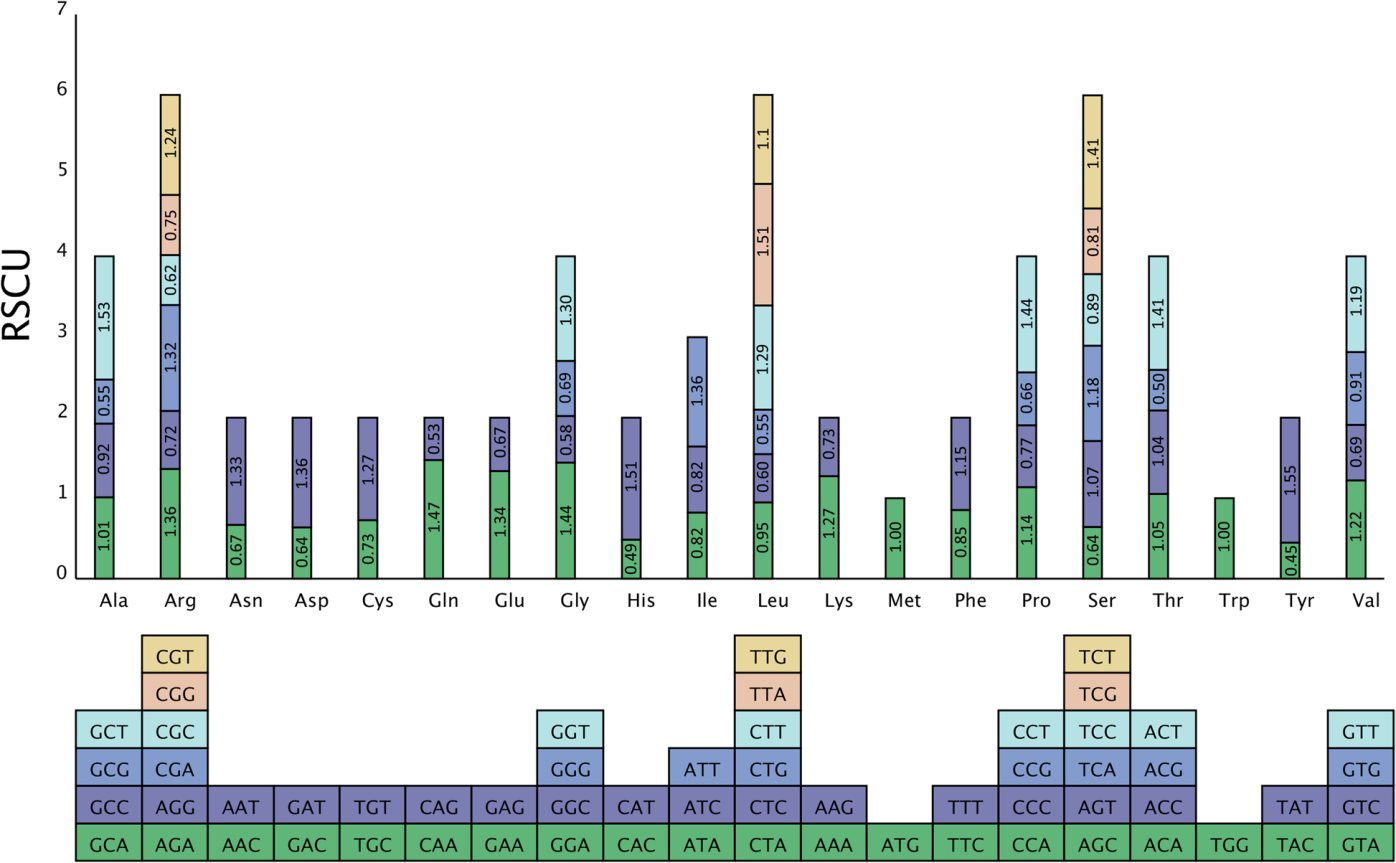
Relative synonymous codon usage (RSCU) in the *B. chinense* mt genome. The different amino acids are shown on the x-axis. RSCU values are the number of times a particular codon is observed relative to the number of times that codon would be expected for a uniform synonymous codon usage

We used self-coded Perl script to analyze the codon composition of the *B. chinense* mt genome. The results are shown in Table 5, the number of codons in all coding genes was 12,704, and the GC1, GC2, and GC3 content and the average GC content of 3 bases (all GC) in the *B. chinense* mt genome were less than 50%, indicating that the codons of the *B. chinense* mt genome were biased because of the use of both A and T bases. The effective codon number (Nc) was 55.48, which is indicative of the weak codon preference of the mt genome. The relative usage of synonymous codons (RSCU) in the *B. chinense* mt genome is shown in Fig. 4. There were 30 codons with RSCU> 1, indicating that the usage frequency of these codons is greater than that of other synonymous codons. Among these, 28 codons ending with the A/T base were identified, and these accounted for 93.33% of the codons, indicating that frequently used codons tend to end with the A/T base.

**Table 5:** Overall characteristics of codon usage in the *B. chinense* mt genome

Parameter	Codon number	GC1	GC2	GC3	GC_all	Nc
value	12,704	48.61	43.45	37.77	43.28	55.48

### Analysis of homologous fragments of mitochondria and chloroplasts

**Fig. 5:**
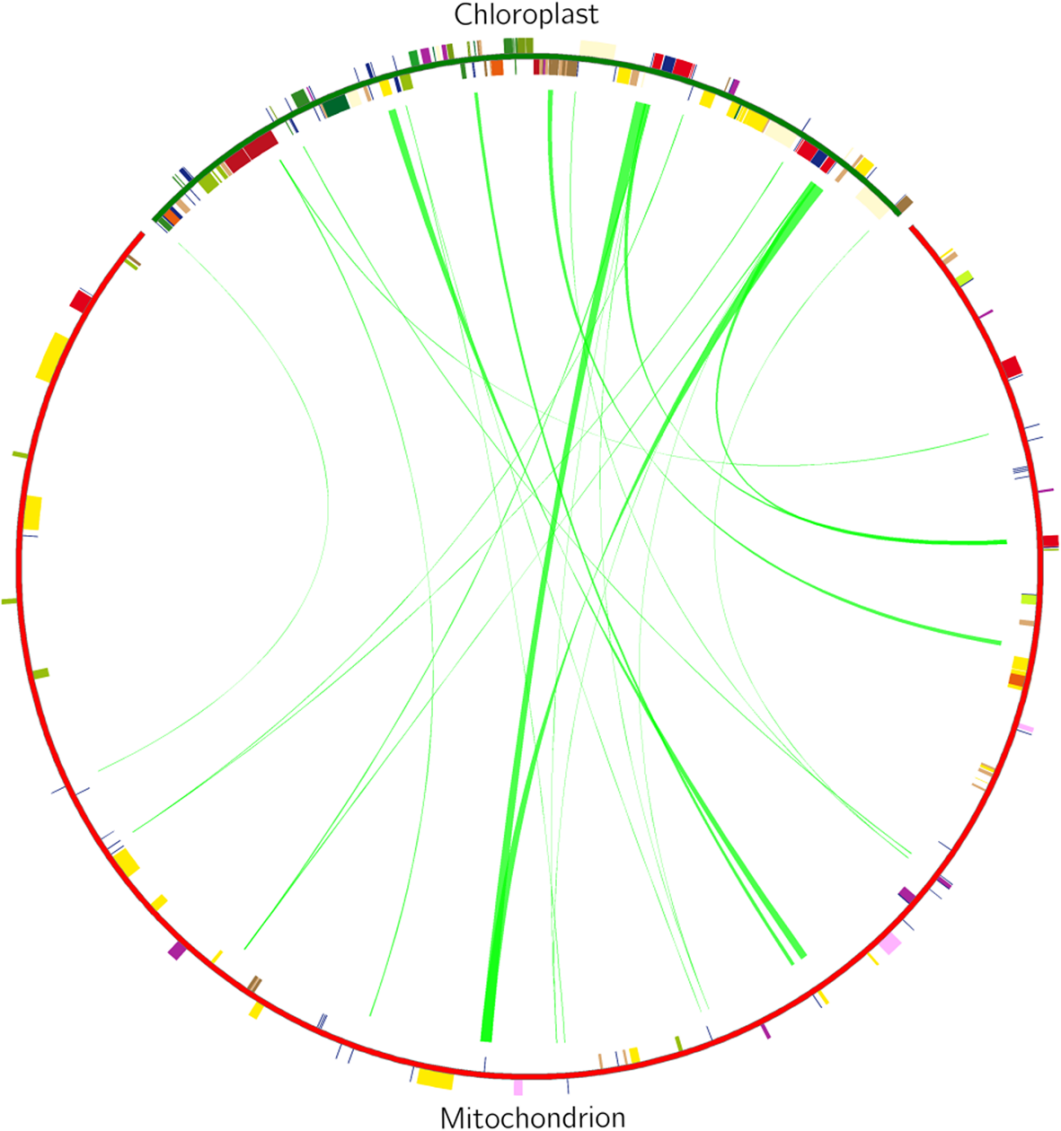
Comparison of a homologous fragment in the *B. chinense* cp genome to that in the mt genome. The green line segment of the circle represents the *B. chinense* cp genome, and the red line segment represents the *B. chinense* mt genome. The green line segment in the circle connects the start and end points of the transferred gene fragments. The width of the green line segment represents the size of the transferred fragment

Using BLAST v2.10.1, we screened the fragments of the *B. chinense* mt genome and cp genome [39] exhibiting > 70% similarity, and performed homologous fragment analysis (Fig. 5). We screened out 25 homologous fragments with a total length of 11,144 bp, which accounted for 2.56% of the mt genome (Table 6). These homologous fragments contained 8 annotated genes, of which 6 were tRNA genes, namely, *trnV-GAC*, *trnW-CCA*, *trnN-GUU*, *trnD-GUC*, *trnM-CAU*, and *trnI-UAU*, and the other two were rRNA gene (*rrn18*) and the cytochrome c biogenesis gene (*ccmC*).

**Table 6:** Comparison of a homologous fragment in the *B. chinense* cp genome to that in the mt genome

	identity/%	Length(bp)	Mismatches	gap opens	mt start	mt end	cp start	cp end	mt gene	cp gene
1	100	2199	0	0	228,120	225,922	99,756	101,954	*trnV-GAC*	*rrn16*/*trnV-GAC*/*ycf15*
2	100	2199	0	0	225,922	228,120	139,185	141,383	*trnV-GAC*	*rrn16*/*trnV-GAC*/*ycf15*
3	86.822	903	96	13	84,048	84,936	82,223	83,116	–	*rpl14*/*rpl16*
4	77.92	1481	235	44	159,455	160,882	49,996	51,437	–	*ndhC*/*ndhJ*/*ndhK*
5	81.343	670	65	36	163,167	162,523	67,474	68,108	*trnW-CCA*	*petG*/*trnP-UGG*/*trnW-CCA*
6	100	240	0	0	279,182	278,943	102,429	102,668	–	*rrn16*
7	100	240	0	0	278,943	279,182	138,471	138,710	–	*rrn16*
8	94.366	213	12	0	42,348	42,560	26,183	26,395	–	*rpoB*
9	94.366	213	12	0	250,603	250,815	26,183	26,395	–	*rpoB*
10	74.099	888	177	38	64,661	63,803	138,729	139,592	*rrn18*	*rrn16*
11	74.099	888	177	38	63,803	64,661	101,547	102,410	*rrn18*	*rrn16*
12	95.614	114	5	0	311,442	311,329	131,516	131,629	*trnN-GUU*	*trnN-GUU*
13	95.614	114	5	0	311,329	311,442	109,510	109,623	*trnN-GUU*	*trnN-GUU*
14	85.632	174	22	3	130,537	130,364	31,634	31,804	*trnD-GUC*	*trnD-GUC*
15	100	78	0	0	325,406	325,329	100	177	*trnH-GUG*	*trnH-GUG*
16	92.308	78	6	0	183,473	183,396	53,659	53,736	*trnM-CAU*	*trnM-CAU*
17	92.308	78	6	0	211,255	211,332	53,659	53,736	*trnM-CAU*	*trnM-CAU*
18	88.312	77	7	2	131,510	131,434	87,592	87,666	*ccmC*/*trnI-UAU*	*trnI-CAU*
19	88.312	77	7	2	131,434	131,510	153,473	153,547	*ccmC*/*trnI-UAU*	*trnI-CAU*
20	97.368	38	1	0	181,820	181,783	102,705	102,742	–	*rrn16*
21	97.368	38	1	0	181,783	181,820	138,397	138,434	–	*rrn16*
22	97.368	38	1	0	212,945	212,908	138,397	138,434	–	*rrn16*
23	97.368	38	1	0	212,908	212,945	102,705	102,742	–	*rrn16*
24	100	34	0	0	311,355	311,322	109,513	109,546	–	*trnN-GUU*
25	100	34	0	0	311,322	311,355	131,593	131,626	–	*trnN-GUU*

The dashes means empty

### Substitution rates of protein-coding genes

**Fig. 6:**
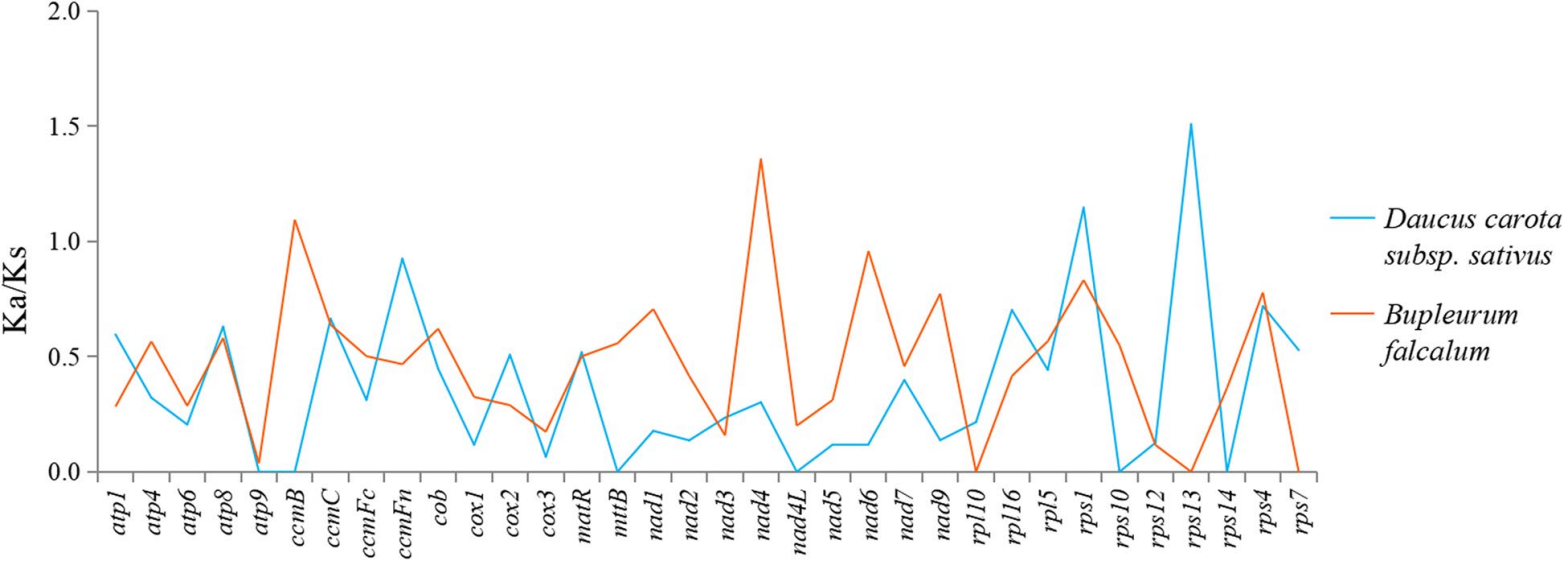
Ka/Ks values of 18 protein-coding genes of *B. chinense* versus those of ten species

It is important to determine the number of non-synonymous substitutions (Ka) and synonymous substitutions (Ks) as it is of great significance for the phylogenetic reconstruction of related species and for understanding the evolutionary dynamics of protein-coding sequences [40, 41]. The Ka/Ks value can be used to determine whether a specific protein-coding gene was under selection pressure during evolution. In the case of a neutral selection, Ks = Ka or Ka/Ks = 1. If the Ka value is higher than the Ks value, it is indicative of positive selection (Ka/Ks > 1), while if Ks > Ka or Ka/Ks < 1, it is indicative of negative selection [42]. The 18 protein-coding genes from the *B. chinense* mt genome were compared with the mt genomes of *Daucus carota* (NC017855) [43] and *B. falcalum* (NC035962) and analyzed using Ka/Ks values. As shown in the Fig. 6, upon comparing the mt genome of *B. chinense* with that of *B. falcalum*, the Ka/Ks value of protein-coding genes such as *ccmB* and *nad4* was found to be > 1, indicating that positive selection had occurred during evolution. In comparison to *D. carota*, the Ka/Ks values of *rps1* and *rps14* were > 1, indicating that both the coding genes in the *B. chinense* mt genome had been positively selected. The Ka/Ks value was < 1 for most protein-coding genes, which indicates that these genes had undergone negative selection during evolution.

### Comparison of the mt genome size and GC content between *B. chinense* and other species

**Fig. 7:**
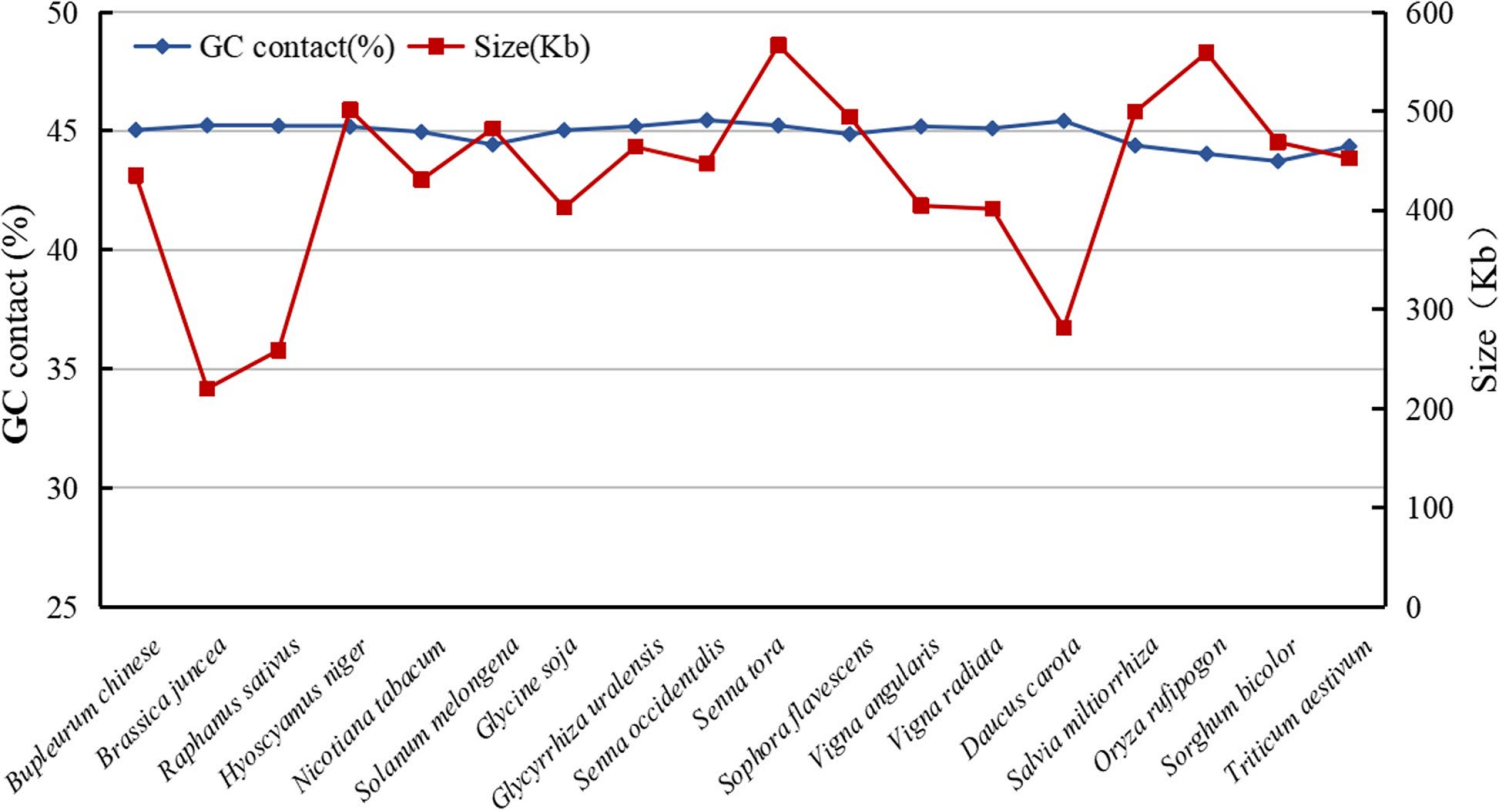
Sizes and GC contents of 18 plant mt genomes

The main characteristics of plant organelle genomes are their genome size and GC content. Seventeen plant mt genomes were selected and their sizes and GC contents were compared with those of the *B. chinense* mt genome. These 17 plant species included 2 species of Cruciferae, 3 species of Solanaceae, 7 species of Leguminosae, 1 specie of Umbelliferae, 1 specie of Labiatae, and 3 species of Gramineae. The species names and accession numbers are shown in Table 7. As shown in Fig. 7, plant mt genome sizes varied greatly, and the size of the selected plant mt genomes ranged from 219.766 Kb (*Brassica juncea*) to 566.589 Kb (*Senna tora*). The difference in the GC content of mt genomes was relatively small, with both being approximately 45%, which indicates that although the size of plant mt genomes differs greatly, their GC content is relatively conserved during the evolutionary process.

**Table 7:** NCBI accession numbers of mt genomes used in this study

Species	Family	Category	Accession number
*Bupleurum chinense*	Umbelliferae	*Bupleurum* L.	OK_166971
*Brassica juncea*	Cruciferae	*Brassica* L.	NC_016123
*Brassica napus*	Cruciferae	*Brassica* L.	NC_008285
*Raphanus sativus*	Cruciferae	*Raphanus* L.	NC_018551.
*Hyoscyamus niger*	Solanaceae	*Hyoscyamu*s L.	NC_026515
*Nicotiana tabacum*	Solanaceae	*Nicotiana* L.	NC_006581
*Solanum melongena*	Solanaceae	*Solanum* L.	NC_050334
*Glycine soja*	Leguminosae	*Glycine* Willd.	NC_039768
*Glycyrrhiza uralensis*	Leguminosae	*Glycyrrhiza* L.	NC_053919
*Senna occidentalis*	Leguminosae	*Cassia* L.	NC_038221
*Senna tora*	Leguminosae	*Cassia* L.	NC_038053
*Sophora flavescens*	Leguminosae	*Sophora* L.	NC_043897
*Vigna angularis*	Leguminosae	*Vigna* Savi	NC_021092
*Vigna radiata*	Leguminosae	*Vigna* Savi	NC_015121
*Daucus carota*	Umbelliferae	*Daucus* L.	NC_017855
*Salvia miltiorrhiza*	Labiatae	*Salvia* L.	NC_023209
*Oryza rufipogon*	Gramineae	*Oryza* L.	NC_013816
*Sorghum bicolor*	Gramineae	*Sorghum* Moench	NC_008360
*Triticum aestivum*	Gramineae	*Triticum* L.	NC_036024
*Ginkgo biloba*	Ginkgoaceae	*Ginkgo* L.	NC_027976

### Phylogenetic analysis

**Fig. 8:**
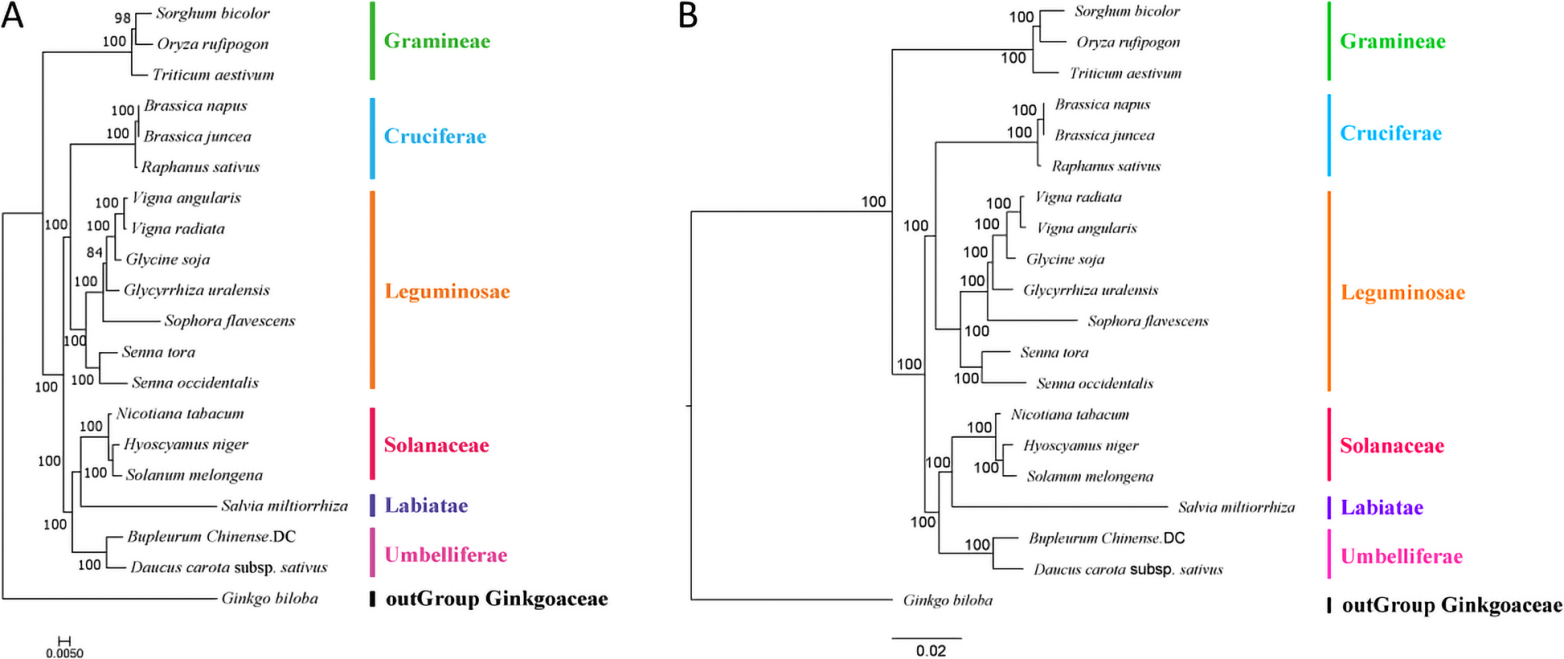
The phylogenetic relationships of *B.chinense* with other 19 plant species. *Ginkgo biloba* served as outgroup. **A** Phylogenetic trees constructed using the maximum-likelihood method. **B** Phylogenetic tree constructed using a Bayesian method

In order to understand the process of evolution of the *B. chinense* mt genome, this article conducted a phylogenetic analysis of the *B. chinense* mt genome and the published mt genomes of 19 plants. Phylogenetic trees were constructed based on maximum likelihood and Bayesian analysis, respectively. The names of the selected species and their NCBI accession numbers are shown in Table 7. The phylogenetic analysis selects *Ginkgo biloba* as an outgroup, the classification results of the phylogenetic tree constructed based on the two analysis methods are consistent. The results showed that Cruciferae, Solanaceae, Leguminosae, Labiatae, Gramineae, and Umbelliferae were well-clustered (Fig. 8). The clustering in the phylogenetic tree is consistent with the relationships of these species at the family and genus levels, indicating that mt genome-based clustering results are reliable. Based on the phylogenetic tree, results were obtained. 20 species of plants were found to be clustered into 3 major groups. *Brassica napus*, *B. juncea*, and *Raphanus sativus*, which belonged to the Cruciferae family, *Vigna angularis*, *V. radiata*, *Glycine soja*, *Glycyrrhiza uralensis*, *Sophora flavescens*, *S. tora* and *S. occidentalis*, which belonged to the Leguminosae family, were grouped together. *B. chinense* and the Umbelliferae plant *D. carota* were clustered into a small group, and the relationship between them was the closest, then clustered with *Hyoscyamus niger*, *Nicotiana tabacum*, *Solanum melongena* and *Salvia miltiorrhiza* into the second group; *Oryza rufipogon*, *Sorghum bicolor*, and *Triticum aestivum*, which belonged to the Gramineae family, were clustered into the third category.

## Discussion

Mitochondria provide plant cells with the energy needed for life processes. Plant mitochondria have a relatively complex genome [44] that exhibits abundant sequence-related changes. They have multiple types of repetitive sequences and relatively conserved coding sequences [45, 46]. The rapid development of genome sequencing technology has accelerated the study of the mitochondrial genome. Our study describes the basic characteristics of the *B. chinense* mt genome for the first time and our findings provide an important basis for understanding the function, inheritance, and evolutionary trajectory of the mt genome. The *B. chinense* mt genome is a circular sequence with a length of 435,023 bp and 45.04% GC content. We performed BLAST analysis and annotated sequences using software, and found that there were 39 protein-coding genes, 35 tRNA genes, 4 rRNA genes, and 2283 ORFs in the mt genome. GC content is a significant factor for assessing species. The GC content of the *B. chinense* mt genome is 45.68%, which is comparable to other sequenced plant mt genomes (*D. carota*, 45.42% [43]; *B. juncea*, 45.24% [15]; *S. flavescens,* 44.86% [47]), but higher than the *B.chinense* cp genome(37.68%) [39]. Since sequence repetitions can cause intermolecular recombination in mitochondria, it is particularly important to perform repetitive sequence analysis [48]. Repetitive sequences in the *B. chinense* mt genome, including simple repetitive sequences, scattered repetitive sequences, and tandem repetitive sequences, were analyzed. The results showed that the *B. chinense* mt genome contained abundant repetitive sequences, and 400 SSR loci were detected; among these, the number of single nucleotide repetitions was the largest. The identified SSRs were mainly composed of the A and T bases. Since the A and T bases were connected via two hydrogen bonds, the energy required to break the bonds is much less than that for the GC bonds and will change more easily. Kuang [49] and Qian [50] have shown that due to the bonds between A and T, SSRs containing AT repeat motifs are more likely to appear in the cp genome as well as in the mt genome. In addition, 844 scattered repeats with a length greater than or equal to 30 bp were identified, and 10 tandem repeats with a greater than 95% match were found.

RNA editing occurs during a post-transcriptional process in the cp and mt genomes of higher plants, and can alter the genetic information at the mRNA level, which enables more efficient protein folding [51]. In this study, 517 RNA editing sites were identified in 34 coding genes of the *B. chinense* mt genome, with a total of 31 codon transfer types. Among the codon transfer types, TCA = > TTA was the most common, with 77 editing sites. After RNA editing, 8.12% of hydrophobic amino acids became hydrophilic, and 48.16% of hydrophilic amino acids became hydrophobic. Consistent results exists in the *Diospyros oleifera* mt genome [52], where the most abundant transfer type in this plant was TCA = > TTA, number 78, which has been edited to change the hydrophobicity of more than half of the amino acids [52]. The selection of *B. chinense* mt genome editing sites showed a strong bias, with all editing sites being C-T editing, which is the most common editing type in plant mt genomes according to several studies [53, 54]. In previous studies, RNA edits that occurred at the second position of a codon accounted for more than half of the total [20, 55]. In the *B. chinense* mt genome, 65.18% of the editing sites were also located at the second-position base of the triplet codon, whose result is consistent with those of previous studies. In addition, after RNA editing, the encoded amino acid will change into stop codons (TAA, TAG, TGA). In the *B. chinense* mt genome, 0.97% of the amino acid is edited into a stop codon, which resulted in the coding process being stopped prematurely, thus altering the function of the gene.

The transfer of plant DNA between organelles and nuclear genomes as well as between species occurs frequently, and sequencing analysis has led to the discovery of DNA transfer events between different genomes (mitochondrial, nuclear and chloroplast) in many plants [56, 57]. Previous studies found that DNA transfer events is mainly organelle genome to nuclear genome DNA fragment transfer, followed by the nuclear genome and plastid genome to mitochondrial genome transfer [58, 59]. Plant mt DNA transfers its sequence fragments to nuclear DNA (rarely to cp DNA), and integrates some nuclear and cp DNA sequences [60, 61]. In high plants, the total length of transferred DNA varies depending on the plant species, lengths ranging from 50 kb (*Arabidopsis thaliana*) to 1.1 Mb (*Oryza sativa* subsp. *Japonica*) [62]. In this study, a total length of 11,144 bp was found to be transferred from the cp genome to the mt genome, accounting for 2.56% of the mt genome. The proportion of the transferred fragments in the mt genomes is similar to the previously reported data for *Acer truncatum* (2.36%) [63] and *Salix suchowensis* (2.8%) [64], but lower than *Suaeda glauca* (5.18%) [20]. In the transfer of DNA fragments from the cp genome to the mt genome of angiosperms, the transfer of tRNA genes is the most common [51]. We identified 25 homologous fragments that had been transferred from the cp genome to the mt genome, these homologous fragments contained 8 annotated genes, of which 6 were tRNA genes. This result is similar to those of Ma [63] et al., who found that the transfer fragment of the *A. truncatum* cp genome to the mt genome contained six integrated genes, of which 5 are tRNA genes.

We analyzed the codons of the *B. chinense* mt genome, and determined the values of related parameters, such as GC1s, GC2s, GC3s, GC_all, Nc, and RSCU. Nc values ranged from 20 to 61; if values were closer to 20, it indicated a stronger codon preference and vice versa [65]. The Nc value of the codon of the *B. chinense* mt genome was 55.48, which indicated that the codon preference of the *B. chinense* mt genome was weak. The RSCU value can reflect the ratio of the actual frequency of use of a codon to the theoretical frequency when there is no usage bias; if RSCU = 1, it means that codon usage is unbiased, and if RSCU< 1, it means that the actual frequency of use of the codon is lower than the frequency of use of other synonymous codons, and if it is vice versa, it is higher than the frequency of use of other synonymous codons [66]. The results of the analysis show that there were 30 codons for which the RSCU> 1, and most of these ended with A/T bases.

The results of Ka/Ks analysis of the mt genomes of *B. chinense*, *D. carota*, and *B. falcalum* showed that most of the genes were negatively selected during the evolution process, indicating that the protein-coding genes of the *B. chinense* mt genome are relatively well-conserved. However, the Ka/Ks value of protein-coding genes such as *ccmB*, *nad4*, *rps1* and *rps14* were found to be > 1, indicating that positive selection occurred during the evolution of these coding genes. Other plant mt genomes also have protein-coding genes with Ka/Ks ratios > 1 [64, 67], and a high gene Ka/Ks ratio plays an important role in further studies on gene selection and evolution of species. The size and GC content of the *B. chinense* mt genome were compared with *D. carota* mt genome. It was found that the size of the mt genome differed greatly, but its GC content was relatively conserved during the evolutionary process. In addition, based on the information obtained from the mt genome, a phylogenetic analysis of the *B. chinense* mt genome and the published mt genomes of 19 plant species was performed. In conclusion, the evolutionary relationships among these species are consistent with the topology of the phylogenetic tree, indicating the consistency of traditional and molecular taxonomy.

## Conclusion

In this study, the mt genome of *B. chinense* was sequenced, assembled, and annotated, and the DNA and amino acid sequences of annotated genes were analyzed thoroughly. The *B. chinense* mt genome is circular and 435,023 bp in length. Seventy-eight genes, of which 39 protein-coding genes, 35 tRNA genes, and 4 rRNA genes, were annotated in the mt genome. Then, the repeat sequences, RNA editing process, and codon preferences of the *B. chinense* mt genome were analyzed. Gene transfer between the mt and cp genomes in *B. chinense* was observed via the detection of gene homologous fragments. In addition, our results also show that although plant mt genomes vary greatly in size, their GC content is relatively conserved during the evolutionary process. The results of Ka/Ks analysis, which was based on coding substitutions, show that most coding genes have undergone negative selection, indicating that mt genes were conserved during the process of evolution. This study provides extensive information regarding the mt genome of *B. chinense*. Importantly, it lays the foundation for future research on genetic variation, systematic evolution, and breeding of *B. chinense* using the mt genome.

## Methods

### Plant growth conditions, DNA extraction, and de novo sequencing

*B. chinense* plants were planted in the traditional Chinese medicine resource garden at the School of Life Sciences, Shanxi Agricultural University(Taigu, Shanxi, China). Plants were kept in the dark for 14 d to obtain etiolated *B. chinense* seedlings. The material was scrubbed with 70% alcohol to remove the dust and soil from the surface of *B. chinense*, snap-frozen in liquid nitrogen, and placed in a pre-cooled 50-mL sealed bag. We collected about 20 g of etiolated *B. chinense* seedlings, transported them using dry ice, and transferred them to the GENEPIONEER laboratory (Nanjing, China). Mt DNA of *B. chinense* was extracted from the sample and sequenced using the Nanopore (2000cUV-Vis) sequencing platform. To obtain a high-quality *B. chinense* mt genome, we used fastp (v0.20.0, https://github.com/OpenGene/fastp) software to filter the raw data, and discard the sequencing junction and primer sequences in the reads, filter out reads with an average quality value of less than Q5, and filter out reads for which the number (N) was greater than 5, and obtain high quality reads. The triple sequenced data were filtered using Filtlong v0.2.1 software, and counted using Perl scripts.

### Assembly and annotation of the mt genome

The original tri-generational data were spliced using Canu assembly software [68] to obtain the contig sequence, which was compared to the plant mt gene database using BLAST v2.6 (https://blast.ncbi.nlm.nih.gov/Blast.cgi). The contig of the mt gene used for comparison was used as the seed sequence, and was extended and cyclized using the original data to determine the master structure (or sub-loop) of the ring; assembly was performed using NextPolish v1.3.1 [69] (https://github.com/Nextomics/NextPolish) using second- and third-generation data. The results were corrected and the results of the final assembly process were obtained after manually checking for errors.

Mt annotation was performed using the following steps: the encoded proteins and rRNAs were compared to published plant mt sequences using BLAST, and further manual adjustments were made based on closely related species. The tRNA was annotated using tRNAscanSE [70] (http://lowelab.ucsc.edu/tRNAscan-SE/). ORFs were annotated using Open Reading Frame Finder (http://www.ncbi.nlm.nih.gov/gorf/gorf.html). The mt genome was constructed using OrganellarGenomeDRAW [71] (https://chlorobox.mpimp-golm.mpg.de/OGDraw.html).

### Analysis of repeat sequences

Interspersed repeat sequences were identified using a combination of vmatch v2.3.0 (http://www.vmatch.de/) software and Perl scripts. The minimum length was set to 30 bp, and four types of sequences were identified: forward, backward, reverse, and complementary. Simple repetitive sequence analysis was performed using MISA online software [72] (https://webblast.ipk-gatersleben.de/misa/). We identified 8, 4, 4, 3, 3, and 3 repeats with 1, 2, 3, 4, 5, and 6 bases, respectively, in this analysis. The minimum distance between the two SSRs was set at 100 bp. Tandem repeats with lengths > 6 bp and > 95% matching repeat units were detected using Tandem Repeats Finder v4.09 software [33] (http://tandem.bu.edu/trf/trf.submit.options.html).

### Analysis of codon composition

The codon composition of the mt genome of *B. chinense* was analyzed using a self-encoded Perl script, to screen for a unique CDS and determine the number of codons per gene, GC content (GC1, GC2, and GC3), average GC content of 3 bases (GC all), effective number of codons (Nc, effective number of codons), and RSCU of synonymous codons.

### Chloroplast to mitochondrion DNA transformation and RNA editing analyses

The cp genome sequence of *B. chinense* (NC_046774.1) [39] was downloaded from NCBI Organelle Genome Resources Database. BLAST v2.10.1 software was used to identify the homologous fragments in the mt genome and cp genome. Screening criteria were set to ensure that the matching rate was ≥70%. The editing sites in the mt RNA of *B. chinense* were identified using the mt gene-encoding proteins of plants as reference proteins. The analysis was conducted using the Plant Predictive RNA Editor (PREP) suite [34] (http://prep.unl.edu/).

### Ka/Ks analysis and phylogenetic tree construction

Synonymous (Ks) and nonsynonymous (Ka) substitution rates of protein-coding genes were analyzed in the mt genome of *B. chinense* using *B. falcalum* and *D. carota* as references. Ka/Ks analysis aligned the CDS sequence using mafft v7.427, Ka/Ks was calculated using the Ka/Ks Calculator v2.0 [42] software MLWL model.

The mt genome sequences of 19 species from different families were aligned using MAFFT [73] software. Connect the aligned sequences end-to-end, trim them with trimAl (v1.4.rev15) (parameter: -gt 0.7), and use jmodeltest-2.1.10 software to predict the model after trimming, and determine that the model is of GTR type. Then use RAxML [74] software, select GTRGAMMA model, bootstrap = 1000, build the maximum likelihood evolutionary tree.

Bayesian analysis was performed using MrBayes v3.2.7, Markov Chain Monte Carlo (MCMC) iterative operation for 1 million generations, sampling every 100 generations. As a result, the initial 25% of the phylogenetic tree is removed (burn-in), and a majority consistent tree is finally obtained.

## Data Availability

The sequence and annotation of *B. chinense* mt genome was submitted to the NCBI. the accession number in Gene Banks is OK166971 (https://www.ncbi.nlm.nih.gov/nuccore/OK166971.1).

## Abbreviations

*B. chinense*: *Bupleurum chinense*
mt: Mitochondria
cp: Chloroplast
